# Patient-reported history of leg ulceration 12–16 years after total primary knee or hip replacement

**DOI:** 10.3109/17453674.2011.596064

**Published:** 2011-09-02

**Authors:** Virginia C Gould, Vikki Wylde, Alison J Smith, Ashley W Blom

**Affiliations:** Musculoskeletal Research Unit, School of Clinical Sciences, Avon Orthopaedic Centre, University of Bristol, Southmead Hospital, Bristol, UK.; Correspondence Ashley.Blom@bristol.ac.uk

## Abstract

**Background and purpose:**

Deep vein thrombosis is common after total joint replacement. It is frequently asymptomatic, and it is unclear whether this leads to longer-term problems such as post-thrombotic syndrome and leg ulceration. We investigated whether the postoperative prevalence of ulceration in patients who had undergone primary total hip replacement (THR) or total knee replacement (TKR) was higher than that found in a control group who had not undergone total joint replacement.

**Methods:**

The study group consisted of patients who had undergone THR or TKR at one orthopedic center 12–16 years previously without routine chemothromboprophylaxis, and who had not undergone revision surgery. The control group was recruited via primary care. All participants were recruited by post and asked to complete a questionnaire. Age- and sex-adjusted prevalence of self-reported leg ulceration was calculated, and logistic regression was used to determine whether there were any associations between THR or TKR and leg ulceration.

**Results:**

Completed questionnaires were received from 441 THR patients (54% response rate), 196 TKR patients (48%) and 967 control participants (36%). No statistically significant differences in age- and sex-adjusted prevalence of ulceration were found between the groups, for either lifetime prevalence or prevalence over the previous 15 years.

**Interpretation:**

Patients who undergo THR and TKR without chemothromboprophylaxis are unlikely to be at a higher risk of long-term venous ulceration than the normal population.

Deep vein thrombosis (DVT) is common after total hip replacement (THR) and total knee replacement (TKR) (Geerts et al. 2001). This is frequently asymptomatic, and it is unclear whether there are any longer-term implications of DVT identified only by venography, in terms of patient morbidity such as post-thrombotic syndrome (PTS) (Cordell-Smith et al. 2004). Chemothromboprophylaxis reduces rates of DVT, but there is no evidence that it reduces deaths from pulmonary embolism (PE) or deaths for any reason (Atkins 2010). In fact, it has been suggested to increase mortality after THR and TKR (Sharrock et al. 2008). This would be difficult to test in a randomized controlled trial, as postoperative mortality is very low after joint replacement ( Parry et al. 2008, Cusick and Beverland 2009).

PTS is a collection of symptoms including swelling, pain, and—in severe cases—venous ulceration. The symptoms of PTS are not uniformly defined, and it is therefore difficult to compare results from different studies (Kahn 2009). It is unclear whether patients who have had TKR or THR are more at risk of PTS than the general population, and whether there are long-term consequences of asymptomatic DVT in this group. Debate about whether to use routine chemothromboprophylaxis is centered mainly on postoperative DVT and pulmonary embolism (Atkins 2010), but the long-term consequences of thrombus are also being increasingly considered (Campbell et al. 2010). This is important, both in the choice of thromboprophylaxis and in ensuring that patients are informed about all potential adverse effects of joint replacement.

Ulceration has been used previously as a “hard” endpoint of PTS to investigate medium-term outcomes of TKR in a study that found no association between postoperative DVT identified by venography and ulceration 5 years after surgery (Muller et al. 2001). Although in that study the prevalence of leg ulceration was considered to be similar to the estimated prevalence of ulceration in the general population, both PTS and leg ulceration are found frequently in the general population (Kahn 2006), and it is difficult to determine whether TKR and THR patients are at an increased risk without a control group.

In this study, we built on the work performed by Muller et al. 2001) by looking at the 12–16-year postoperative prevalence of leg ulceration in both TKR and THR patients. The inclusion of a control group allowed comparison of the prevalence of leg ulceration to that of the general population, in order to identify whether THR or TKR is indeed a risk factor for leg ulceration.

## Patients and methods

Between 1993 and 1996, 956 patients underwent primary unilateral TKR and 1,727 patients underwent primary unilateral THR at one elective orthopedic center (Blom et al. 2003, 2006). These patients received no routine chemothromboprophylaxis.

Of these patients, 790 TKR patients and 1,240 THR patients responded to a cross-sectional postal survey in 2001 (Wylde et al. 2009) and they were used as the basis for this study. After excluding deceased and untraceable patients, 410 TKR and 813 THR patients were invited to take part by post, with a single reminder sent to non-responders. In the questionnaire, patients were asked if they had had leg ulcers before their joint replacement, since their joint replacement, and presently, on either the operated leg or the contralateral leg.

Control participants were recruited through GP surgeries in the Bristol area. Healthy controls were defined as individuals who had not had either THR or TKR. Questionnaires were posted to randomly selected patients who were in the same age group as the patients. The control participants were asked in the questionnaire if they had ever had ulcers on either leg, and if so, whether these had occurred within the previous 15 years.

### Statistics

Due to differences in age distribution between the three groups, the lifetime prevalence of ulceration was adjusted by standardizing the age and sex distributions of the THR and TKR groups to that of the controls. 95% confidence intervals (CIs) for proportions were calculated using the Wilson method (Wilson 1927). Logistic regression with CIs was used to determine whether there were associations between THR or TKR and leg ulceration. We used SPSS software version 16.0.

### Ethics

Ethical approval was obtained from the Southmead Research Ethics Committee (08/H0102/62).

## Results

### Response rates

Of the 813 THR patients who were invited to participate, 485 responded to the postal survey. Of the responders, 44 were excluded due to revision surgery or dementia, giving a final total of 441 THR patients (54% response rate). Of the 410 TKR patients who were invited to participate, 220 responded to the postal survey, and of these, 24 were excluded due to revision surgery or dementia, giving a final total of 196 TKR patients (48% response rate). Overall, 2,700 healthy controls were invited to participate, and 978 responses were received; of these, 6 were not eligible due to having had TKR or THR and a further 5 were excluded as they did not specify their ages. There were therefore 967 healthy controls, corresponding to a response rate of 36%.

### Demographics

Before adjustment, the median age of the TKR patients was 81 (33–98) years, that of ofthe THR patients was 78 (30–99) years, and that of the healthy controls was 72 (34–96) years (Table 1).

**Table 1.** Numbers of participants in each group, according to age

		Age (years)							
Group	females	30–39	40–49	50–59	60–69	70–79	80–89	90–100	Total
Control group	56%	14 (1.4%)	17 (1.8%)	123 (13%)	194 (20%)	361 (37%)	211 (22%)	47 (4.9%)	967
THR patients	62%	4 (0.9%)	8 (1.8%)	17 (3.9%)	81 (18%)	150 (34%)	150 (34%)	31 (7.0%)	441
TKR patients	65%	2 (1.0%)	0 (0.0%)	5 (2.6%)	13 (6.6%)	68 (35%)	88 (45%)	20 (10%)	196

The median time from surgery until follow-up was 14 (12–16) years for both TKR patients and THR patients. The contralateral joint had also been replaced in 72% of the TKR patients and in 53% of the THR patients.

For the THR patients, the median age of responders to this study at the time of surgery was 64 (17–86) years, as compared to a median age of 69 (17–91) years for all patients who underwent primary THR at the center 12–16 years previously. For TKR patients, the median age of responders at the time of surgery was 67 (19–86) years, as compared to a median age of 72 (19–90) years for all patients who underwent primary TKR at the center 12–16 years previously. However, given the long-term nature of this study, it was to be expected that there would be loss of potential participants due to morbidity and mortality, and that this would be seen particularly in the more elderly patients. Sensitivity analysis was performed to identify non-response bias. When analysis was adjusted to standardize the age and sex distribution of responders to that of all those invited to participate, the results did not change statistically significantly.

### Lifetime prevalence of ulceration

The age- and sex-adjusted lifetime prevalence of ulceration, open and healed, in the 3 groups was similar (Table 2). The unadjusted lifetime prevalence of ulceration was 7.6% (CI: 5.4–11) for the THR group and 12% (7.7–17) for the TKR group.

**Table 2.** Lifetime prevalence of ulceration (adjusted for age and sex)

	Prevalence	95% CI
Control participants	7.4%	5.9–9.2
THR patients	5.9%	4.0–8.6
TKR patients	9.3%	6.0–14

### Postoperative prevalence of ulceration

For the purposes of analysis, ulceration within the previous 15 years in the control group was considered equivalent to postoperative ulceration in the study group. The prevalence of ulceration was highest in the TKR group, with considerable overlap in the confidence intervals (Table 3). The unadjusted postoperative prevalence of ulceration was 5.7% (CI: 4.1–8.6) for the THR group and 9.9% (6.4–15) for the TKR group.

**Table 3.** Prevalence of ulceration postoperatively (patients) or in the previous 15 years

	Prevalence	95% CI
Control participants	5.7%	4.4–7.4
THR patients	4.7%	3.1–7.4
TKR patients	7.8%	4.8–12

### Risk of ulceration

Logistic regression did not show any evidence that patients who had had a THR and TKR were at an increased risk of leg ulceration postoperatively, compared to healthy controls (Table 4). The 95% confidence intervals for the odds ratios contained the value of 1, indicating that it is plausible at the 0.05 level that those who had had a THR or a TKR were as likely to develop a leg ulcer as those in the control group. However, increasing age was found to be associated with increased risk of leg ulceration.

**Table 4.** Logistic regression analysis

	Odds ratio	95% CI	p-value
THR	1.2	0.7–1.9	0.5
TKR	0.8	0.4–1.4	0.4
Sex	0.7	0.5–1.1	0.1
Age	1.0	1.0–1.1	< 0.001

## Discussion

It has been reported previously that there is no increased risk of venous ulceration 5–12 years after THR, or 5 years after TKR, when compared to an estimated incidence in the general population (Muller et al. 2001, Mehta et al. 2003). This study expands on these previous findings, both by extending the time period of the study and by including a control group who had not undergone THR or TKR. We found no association between having had a TKR or a THR and having leg ulceration by 12–16 years after surgery.

Previous studies have investigated differences between patients who have had postoperative DVT identified by venography and those who have not had DVT. An association between asymptomatic DVT and later development of PTS was identified in a meta-analysis, which included both abdominal and joint surgery, but the study was limited by a lack of consistency in methods and definitions between the few papers published on the subject (Wille-Jorgensen et al. 2005). 1 year after surgery, no difference was found in morbidity or patient outcome between THR and TKR patients who had had a postoperative DVT identified by venography and those who had not (Cordell-Smith et al. 2004); nor was DVT that was diagnosed by venous ultrasonography postoperatively—whether symptomatic or not—found to be predictive of PTS in a group of TKR patients 1–7.5 years after surgery (McAndrew et al. 2010), or associated with the incidence or prevalence of leg ulcers 5 years after TKR (Muller et al. 2001). Mant et al. (2008) found a low frequency of PTS 2–4 years after THR in patients who received had chemothromboprophylaxis; however, there was no control group that did not receive thromboprophylaxis.

Kim and Kim (2002) and Kim et al. (2003) reported that DVT was found in 39–45% of patients after TKR and in 20–27% after THR; at 6-month follow-up, it was found that these thrombi had all spontaneously and completely resolved. It is likely that the patients in our study would have had similar rates of asymptomatic DVT.

The patients in the study group were asked about ulceration before and after their surgery, while individuals in the control group were asked if they had had an ulcer within the previous 15 years or more than 15 years ago. We recognize that recall of the timing of events that long ago is unlikely to be accurate, and that there are also problems with self-reporting of ulceration. In a previous study, of 43 patients who reported having had ulceration, 12 did not have true venous ulceration (Mehta et al. 2003). It is likely that the true incidence of ulceration is lower than that reported in our study. However, the control group and patient group data were obtained from using almost identical questions, thus allowing direct comparison between the groups.

In this study, we found no evidence that patients who underwent THR or TKR without chemothromboprophylaxis were at higher risk of venous ulceration than the normal population by 12–16 years postoperatively. However, a larger sample size would be needed to enable detection of a small difference with any degree of statistical confidence. Recommendations for further work would include a large-scale prospective cohort study to assess the long-term incidence of venous ulceration in joint replacement patients with confirmed postoperative DVT and in those without DVT.
